# Ye Festive Bolter

**Published:** 1886-03

**Authors:** 


### "YE FESTIVE BOLTER."

"There can be no doubt," says the pungent *Louisville Medical Herald*, "as to who are pulling wires,—'the fine Italian hand' of '*ye festive bolter*' is plainly visible in the misty background. These malcontents propose to go to St. Louis next May, with a cut and dried delegation, where they expect to *swallow* the entire American Medical Association, and then place the "Old Code" on top of that *august body* to keep it down. We warn our friends to remember the old maxim that 'the price of liberty is eternal vigilance;' and that 'forewarned is forearmed.' *Do your duty*, and the result will be all you desire."

We say amen to the above. At New Orleans the American Medical Association, almost to a man, were generous enough to acquit the Billings-Hays-Committee of *intentionally* transcending their instructions and authority in proceeding, as they did, to organize the International Medical Congress—though none of them could help feeling a sickening sense at the modesty (?) with which they appointed *themselves*, *first*, to everything they could absorb;—but we are satisfied now, especially in the light of recent developments, that it was a case of misplaced confidence, and that the assumption of the authority to make *any* appointments, or to do *anything* beyond deliver the invitation of the A. M. A. to the Congress, and report back "yes or no," was deliberate and intentional,—a bold attempt to bulldoze and control the profession of America and the Association. And, moreover, they spurned the Association (whence they got their existence and *any* power,)—most contemptuously; an assumption of leadership, which, in their arrogance, they never *dreamed* anybody would question, much less *object* to; and when *Texas* had the temerity to mildly protest that *all* the civilization of America is not in the New England States; and that the American Medical Association, being a representative body, the Congress should be also—those gentlemen were never more surprised;—and when it was seriously proposed to (and was done,) appoint a member from each State, to be added to their immaculate committee, with instructions to take off all those New Code names—make the committee disgorge some of the offices they had swallowed, and scatter the balance a little more evenly, and equitably amongst the *representation* in the A. M. A., behold! they get mad, and accuse Texans and others who had been so outraged, of being a lot of unknown nobodies—a lot of schemers and plotters who "captured" the Association! That is more like the thief joining in the cry "stop thief" than anything else. Then these immaculate seven wise men disdained to affiliate with their co-committee-men from the States, and resigned! The rest is known too well to repeat. The committee which was thus left alone in possession of the field—after having organized, or nearly organized, the Congress—did a very foolish and weak thing; they *begged* these immaculates to come back; and got snubbed for their pains;—good! Now the demand is,—the ultimatum presented for the consideration of the profession of America—that they will all step aside, acknowledge their imbecility, their utter incapacity to organize anything, and let the i. s. w. m. (that means the immaculate seven wise men), whom the pungent *Louisville Medical Herald* so irreverently calls "*ye festive bolters*" step to the front, put all the new code men back on the list, reabsorb their quota of the principal offices,—and *restrict* the representation in the Congress to God's country;—in short, to make us—all of us—" eat crow! This, gentlemen delegates to St. Louis, is what you are to expect, and then, if you do not consent,—they will try *first* to outvote you with their packed delegates—the Association and the Code are to be destroyed—busted up—as being too democratic! The fact is, these gentlemen do not care a button for the new code men, whether they are appointed to the Congress or not;—that is only a pretext, as we have said elsewhere, for making vigorous war on the Code—the Code is the burr that sticks in their craw, and hinders the swallowing of consultations with quacks—and that's what's the matter with Hannah!

CONGRATULATIONS.—We extend our congratulations to our young Mississippi friend, Dr. E. A. Nealy, who, having graduated with first honors, and delivered the valedictory also, at the Memphis Medical College, is made Associate Editor of the *Mississippi Valley Medical Journal.*

